# Thermal asymmetry and density-dependent behavioural shifts drive competitive displacement between two globally invasive ants

**DOI:** 10.1098/rspb.2026.1951

**Published:** 2026-11-11

**Authors:** Defu Chen, Zhuoquan Liao, Jiale Song, Zengyuan Ke, Mengfei Mao, Shaopeng Li, Yongyue Lu, Yijuan Xu

**Affiliations:** ^1^ Red Imported Fire Ant Research Center, South China Agricultural University, Guangzhou 510642, People's Republic of China; ^2^ School of Ecological and Environmental Sciences, East China Normal University, Shanghai 200241, People's Republic of China

**Keywords:** co-invasion, invasional meltdown hypothesis, interference competition, behavioural plasticity, ecological release, solenopsis invicta, wasmannia auropunctata

## Abstract

While co-occurring invasive species are often assumed to accelerate degradation via ‘invasional meltdown,’ the temporal dynamics of their interactions and cascading effects on native communities remain poorly resolved. Here, we investigate the spatiotemporal interactions between two devastating invasive ants, *Solenopsis invicta* and *Wasmannia auropunctata*. Through habitat snapshots, year-long monitoring and behavioural assays, we show that co-invasion yields highly dynamic, asymmetric temporal displacement rather than synergistic meltdown. Initially, intense antagonism between the invaders generates a transient ecological buffer that shields native ants from exclusion, departing from additive null model expectations. Over time, however, this buffer decays as *W. auropunctata* progressively displaces *S. invicta*, precipitating the extirpation of native ants. Mechanistically, this turnover is mediated by divergent temperature responses—*W. auropunctata* responds positively to warming, while *S. invicta* remains unresponsive—coupled with density-dependent behavioural state shifts. When rare, *W. auropunctata* employs thanatosis to survive; upon crossing a density threshold, it transitions to lethal mass attacks to eradicate *S. invicta*. Ultimately, our findings resolve the dynamics of competitive displacement between apex invaders, highlighting the need for dynamic, systemic management of complex co-invasions.

## 1. Introduction

With the rapid expansion of global trade networks, the establishment frequency of non-native species is accelerating at an unprecedented rate, making the sympatric co-occurrence of multiple invasive species within the same ecoregion increasingly ubiquitous [1]. Compounding this trend, global climate change is acting as a potent catalyst for biological invasions. The evidence highlights that shifting thermal regimes not only facilitate the poleward and upward range expansion of non-native species [2], but warming temperatures also disproportionately favour invaders, which often exhibit broader thermal tolerances and superior phenotypic plasticity compared to native taxa [3,4]. Consequently, understanding the combined impacts of co-occurring invaders on native assemblages, and deciphering the critical role of climatic conditions in shaping these complex interactions, have emerged as core issues in modern ecology.

The classical theoretical framework of multiple stressor impacts conceptualizes the interactions between invaders and their ecological consequences into three fundamental categories [5]. Additive effects occur when invasive species operate independently, and their combined impact equals the simple sum of their individual effects [6,7]. For instance, in aquatic food webs, the American bullfrog (*Lithobates catesbeianus*) and the western mosquitofish (*Gambusia affinis*) independently prey on native tree frogs without non-linear interference; their co-occurrence leads to a strictly linear, additive decline in native amphibian survival [8]. Synergistic effects arise when positive interactions between invaders—such as mutualism or habitat modification—exponentially amplify their collective destructiveness. This synergy forms the theoretical bedrock of the ‘invasional meltdown’ hypothesis, which has dominated invasion biology for the past two decades [9,10]. A textbook example is the mutualism between the invasive yellow crazy ant (*Anoplolepis gracilipes*) and honeydew-producing scale insects (*Tachardina aurantiaca*) on Christmas Island, which fuelled the formation of ultra-dense supercolonies that cooperatively eradicated the keystone native red crab and fundamentally altered forest understory and litter dynamics [11]. Beyond such cross-taxa mutualisms, invasional facilitation can also occur at ecosystem scales among taxonomically closely related species via positive feedback loops, such as the grass-fire cycle driven by co-occurring exotic annual grasses [12]. Furthermore, recent empirical evidence highlights that facilitation among co-invaders is often asymmetric and promoted by strategic divergence along the key functional axes; for instance, ‘good competitors’ can reap substantial biomass benefits by exploiting favourable microenvironments shaped by weaker competitors acting as ‘good cultivators’ [13]. Conversely, antagonistic effects occur when intense interspecific competition or mutual interference results in a combined impact less than the sum of their individual expectations [14,15]. For example, the invasion of the warty comb jelly (*Mnemiopsis leidyi*) decimated native zooplankton and collapsed the anchovy fishery in the Black Sea; however, the subsequent accidental introduction of a specialized predatory comb jelly (*Beroe ovata*) imposed extreme top-down antagonistic control, heavily suppressing the former invader and relieving pressure on the native community [16]. In nature, particularly when the apex predators occupying highly overlapping niches encounter one another, intense resource contention often makes antagonism the dominant force structuring communities, as predicted by the competitive exclusion principle [17].

Competitive displacement, defined as the local exclusion of a previously established species owing to direct or indirect competitive interactions with another species [18], is a widespread phenomenon. Across various insect taxa, this process is frequently driven by highly dynamic, asymmetric behavioural or reproductive interferences, such as the evolutionary shifts in satyrization resistance observed in co-existing invasive mosquito lineages [19]. Yet, in multi-species invasions, classical frameworks (additive, synergistic and antagonistic effects) predominantly treat these interactions as static, equilibrium endpoints [15], often relying on single-time-point ecological snapshots that truncate temporal continuity [20]. In reality, biological invasion is a highly time-dependent, complex process [21]. Sympatric antagonism between co-occurring invaders frequently precipitates invasion turnover (or successional replacement), where an initially dominant invader is progressively supplanted by a secondary competitor over time [22,23]. A classic example of such successional dynamics is the landscape-scale displacement of the red imported fire ant (*Solenopsis invicta*) by the invasive tawny crazy ant (*Nylanderia fulva*), driven by specialized behavioural and physiological adaptations [22,24]. Understanding these temporal dynamics is therefore crucial to resolving the long-term trajectories of complex co-invasions.

Invasive ants are among the most destructive non-native taxa globally, serving as ideal model systems for invasion ecology owing to their disruptive impacts on native arthropod networks and ecosystem functioning [25–27]. In southern China, the distribution ranges of two apex predators introduced in the twenty-first century—the red imported fire ant (*S. invicta*) and the little fire ant (*Wasmannia auropunctata*), both listed among the ‘100 of the World's Worst Invasive Alien Species’ [28]—are increasingly overlapping. In their respective invasion histories, both the species are notorious for their massive supercolonies maintained by polygynous and unicolonial reproductive strategies, extreme interference competition capabilities and fatal exclusionary impacts on native arthropod assemblages ([29,30]; Le Breton *et al.* [31,32]). Conventionally, when two dominant competitors with overlapping niches encounter one another, they are expected to either engage in a prolonged, attritional coexistence or drive mutually detrimental competitive exclusion. Yet, their real-world interaction trajectories—particularly the temporal direction of community succession—remain largely elusive.

To bridge this critical knowledge gap, we first conducted ecological snapshot surveys across single-invasion plots of *S. invicta*, single-invasion plots of *W. auropunctata* and their co-invasion plots in Shantou, Guangdong, to determine whether a synergistic relationship exists between the two invasive ants. Subsequently, we established a year-long spatial–temporal continuous monitoring network in Enping, Guangdong. By comparing *S. invicta* single-invaded plots, *S. invicta* and *W. auropunctata* co-invaded plots and non-invaded native communities, we not only tested the classic ‘invasional meltdown’ hypothesis, but also explicitly investigated the processes and underlying mechanisms of competitive displacement. Specifically, this study seeks to answer three core questions: (1) does the sympatric co-occurrence of two apex invasive ants leads to mutually facilitative coexistence (i.e. ‘invasional meltdown’), or does the identity of the dominant invader undergo dynamic turnover over time? (2) How does this dynamic process of multiple invasions reshape the biodiversity trajectory of native ant assemblages? (3) If competitive displacement occurs, what are the underlying ecological mechanisms? By addressing these questions, we aim to uncover the hidden temporal dynamics underlying multiple invasions and provide a novel theoretical framework for predicting and managing increasingly complex global biological invasions.

## 2. Material and methods

### (a) Study sites, ant collection and environmental factors

To investigate the potential interspecific interactions and invasion dynamics among invasive ant species, this study integrated two strategies: a spatial snapshot survey and longitudinal dynamic monitoring. First, in August 2024, a spatial snapshot survey was conducted in Shantou City, Guangdong Province. A total of 48 plots were randomly selected, comprising three uninvaded plots, 15 plots invaded solely by the red imported fire ant (*S. invicta*), 18 plots invaded solely by the little fire ant (*W. auropunctata*), and 12 plots co-invaded by both the species. To ensure spatial independence while capturing landscape-scale variation, the inter-plot distances for the 48 spatial snapshot plots in Shantou ranged from a minimum of 100 m to a maximum of 23 km. All the selected plots shared a similar habitat type—heavily anthropogenically disturbed urban greenbelts. Furthermore, a 1-yr longitudinal dynamic monitoring program was carried out from September 2024 to August 2025 in Enping City, Guangdong Province. For the 10 longitudinal monitoring plots in Enping, inter-plot distances ranged between 100 m and 30 km. This monitoring included 10 plots situated in mixed agricultural and wasteland habitats (five uninvaded, three solely invaded by *S. invicta* and two co-invaded plots). Notably, Enping City was selected as the time-series monitoring site based on our baseline census conducted in 2023 (manuscript under review). The census revealed that this region represents a newly colonized area for *W. auropunctata* with low initial population densities, providing an ideal natural system to study the early dynamics of invasion between the two invasive ants under natural conditions.

Each study plot measured 12 m × 9 m. To comprehensively characterize the local ant community, we employed a standardized sampling strategy combining pitfall trapping [33] and Winkler extraction [34,35]. Specifically, 12 pitfall traps (plastic cups: 4.8 cm bottom diameter, 6.45 cm top diameter, 7 cm height, approx. 175 ml capacity) were deployed in each plot, following a standardized grid layout designed for subtropical invasive ant assessments [36,37]. The rim of each cup was set flush with the soil surface, and the cups were filled to one-third of their volume with a 1 : 1 mixture of absolute ethanol and propylene glycol. Traps were collected after 48 h. Litter collection and Winkler extraction strictly followed the standardized protocols of Lopes and Vasconcelos [35]. Samples collected via both the methods from the same plot were pooled to represent the ant community composition of that specific site.

To control for the potential confounding effects of real-time weather conditions on sampling, daily meteorological data during the sampling periods (including mean temperature, precipitation, relative humidity and atmospheric pressure) were obtained from the China Meteorological Data Service Centre (http://data.cma.cn/).

### (b) Design of indoor asymmetric interference competition experiments

This study posits that during the invasion process, the ability of *W. auropunctata*—a secondary invader—to successfully colonize areas dominated by high-density *S. invicta* is primarily attributable to its unique behavioural strategies. Specifically, during inevitable interference competition, *W. auropunctata* significantly prolongs its survival by exhibiting ‘death-feigning’ (thanatosis) behaviour. To validate this hypothesis from a behavioural mechanism perspective, we designed a series of indoor aggression and survival experiments. The design of these asymmetric confrontation trials and the attack index referred to Nie *et al.* [38].

First, preliminary one-on-one trials were conducted to evaluate behavioural interactions between the two species. For these assays, we collected four independent colonies for each of the three target species (*S. invicta* from Guangzhou; *W. auropunctata* from Enping; *Solenopsis jacoti* from Xiaogan). For the invasive species, sampled nests were separated by at least 20 m for *S. invicta* and 10 m for *W. auropunctata* to ensure sampling from physically distinct nest mounds across the site. However, given that both *S. invicta* and *W. auropunctata* predominantly exhibit supercoloniality and unicolonial social structures in their introduced ranges [25], these physically separate nests likely shared a supercolonial origin. Based on prior research [39] and our preliminary validation, this unicolonial nature intrinsically accounts for the lack of inter-nest behavioural divergence, justifying why colony identity was not treated as a random effect. A single *S. invicta* worker and a single *W. auropunctata* worker were introduced into a Petri dish (5.5 cm diameter). Over a 5-min period (36 replicates), we focused on the decisive outcomes of contact. While intermediate interactions (avoidance, threat displays) were observed, our analysis specifically quantified the frequency of thanatosis to confirm it as a primary defensive node.

Building on these 1v1 observations, we then established asymmetric confrontation trials to test whether this thanatosis strategy translates into a survival advantage under intense ecological pressure. To simulate the early stage of invasion, where *S. invicta* holds an absolute numerical advantage, we introduced different densities (two, four, six or eight workers) of *S. invicta* against one *W. auropunctata*. The survival status of the *W. auropunctata* worker was recorded at 10, 20, 30 and 60 min. We conducted 20 independent replicates per density gradient, divided into four batches (five dishes per batch) to calculate batch mortality rates. To control for body size, the native species *S. jacoti*, which is similar in size but lacks thanatosis, was used in parallel trials following the identical protocol.

Finally, to test the late-stage invasion scenario where *W. auropunctata* achieves numerical superiority, reverse-density validation experiments were conducted. We introduced *W. auropunctata* at densities of two, four, six or eight workers against one *S. invicta* worker, recording the mortality of *S. invicta* over 60 min. *S. jacoti* was again used as a control for *W. auropunctata* to evaluate whether the offensive capacity was unique to the invader. All experimental designs and attack criteria referred to Nie *et al.* [38].

## 3. Results

### (a) Antagonistic interactions and community buffering under co-invasion

In the snapshot survey, both the single invasions of *S. invicta* and *W. auropunctata* triggered severe declines in richness and abundance of native ant communities, yet the magnitude of their impacts diverged significantly. Compared with uninvaded habitats, a single invasion by *S. invicta* led to a sharp decrease in native ant species richness (figure 1A) and abundance (figure 1B). Strikingly, *W. auropunctata* exhibited a far more devastating suppressive effect, driving native ant communities toward near-local extirpation, with both richness and abundance approaching zero (p<0.05).

**Figure 1.**
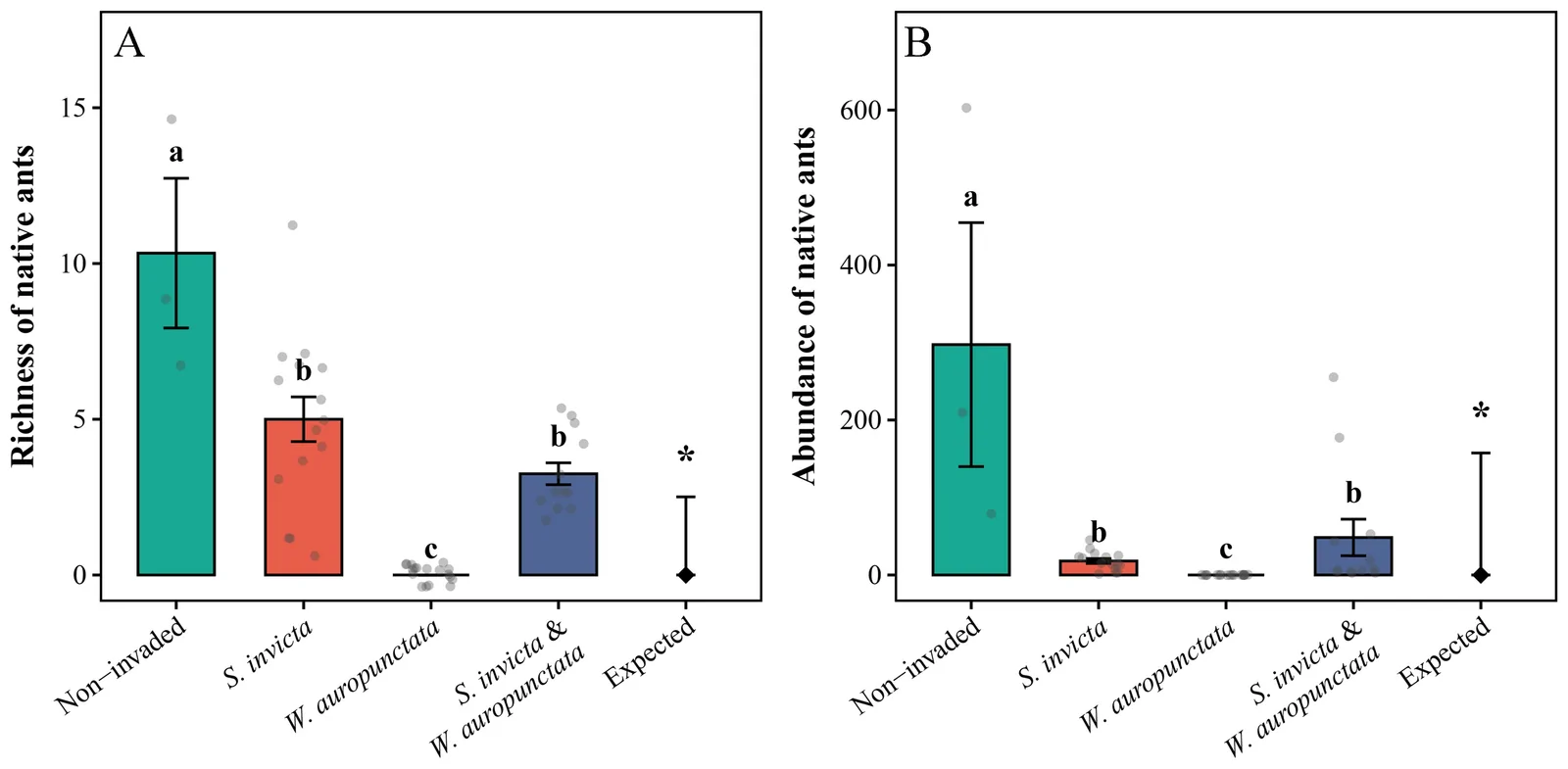
Validation of the antagonistic relationship between *S. invicta* and *W. auropunctata*. (A) Native ant species richness. (B) Native ant abundance. In each subplot, the first four bars represent the observed data, while the predicted data (diamonds denote theoretical central values) were generated based on an additive null model. Small grey dots represent raw data. Different letters indicate significant differences among the observed data, and asterisks denote significant differences between the predicted values and the observed co-invaded areas.

In co-invaded habitats, native ant richness and abundance remained significantly impoverished compared with the uninvaded baseline, yet they were statistically indistinguishable from the levels observed under *S. invicta* single invasion. To mechanistically disentangle the interaction patterns of these two combined invaders, we compared empirical observations from the co-invaded plots against expectations generated by an additive null model. The results demonstrated that the actual observed values for both native ant richness and abundance under co-invasion were significantly higher than the additive expectations (p<0.05). Under a strict additive assumption, the combined suppression of both invasive ants should have completely eradicated the native ants (expected value ≈0). This significant deviation from the additive expectation provides compelling empirical evidence for robust antagonistic interactions between *S. invicta* and *W. auropunctata*. Paradoxically, this antagonism buffered the native community, sparing it from total competitive exclusion.

### (b) Community disassembly and spatio-temporal decline trajectories along invasion gradients

Field observations based on long-term temporal dynamics revealed hierarchical suppressive effects of different invasion scenarios on native ant diversity. Specifically, native ant species richness in co-invaded plots was significantly lower than in both the uninvaded baseline and *S. invicta*-only plots; simultaneously, a significant difference persisted between uninvaded and *S. invicta*-invaded plots (figure 2A). Consistently, under the co-invasion scenario, native ant abundance was suppressed to an extreme minimum (figure 2B). Single-season analyses of native ant richness and abundance echoed similar patterns, consistently showing the lowest values in co-invaded plots (supplementary material, figures S1 and S2). Furthermore, PCoA demonstrated that the native ant community composition underwent profound turnover, exhibiting significant structural divergence across the three distinct invasion scenarios (figure 3).

**Figure 2.**
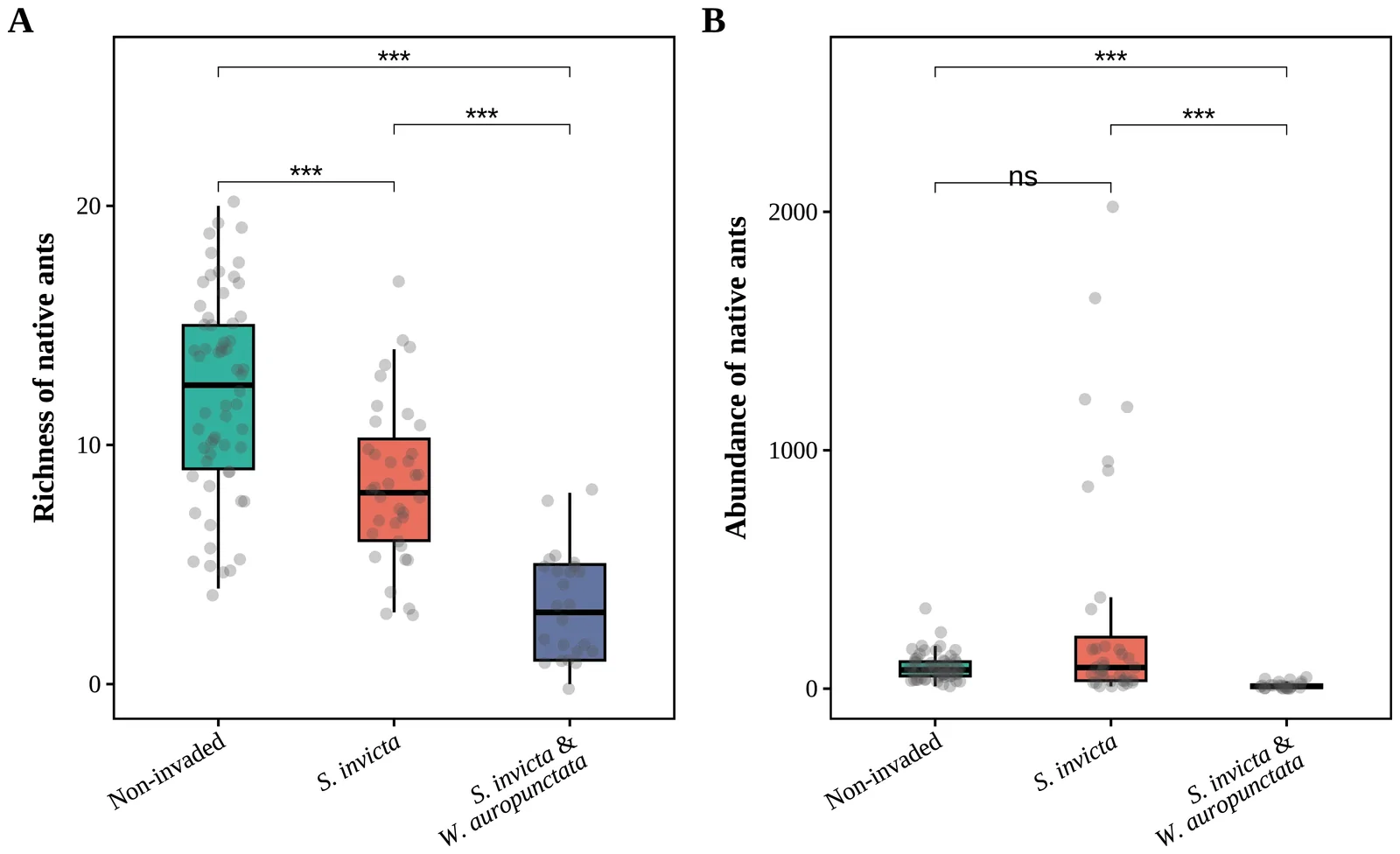
Comparison of native ant species richness (A) and abundance (B) across different invasion plots, based on temporal dynamic data from Enping City. Small grey dots represent raw data.

**Figure 3.**
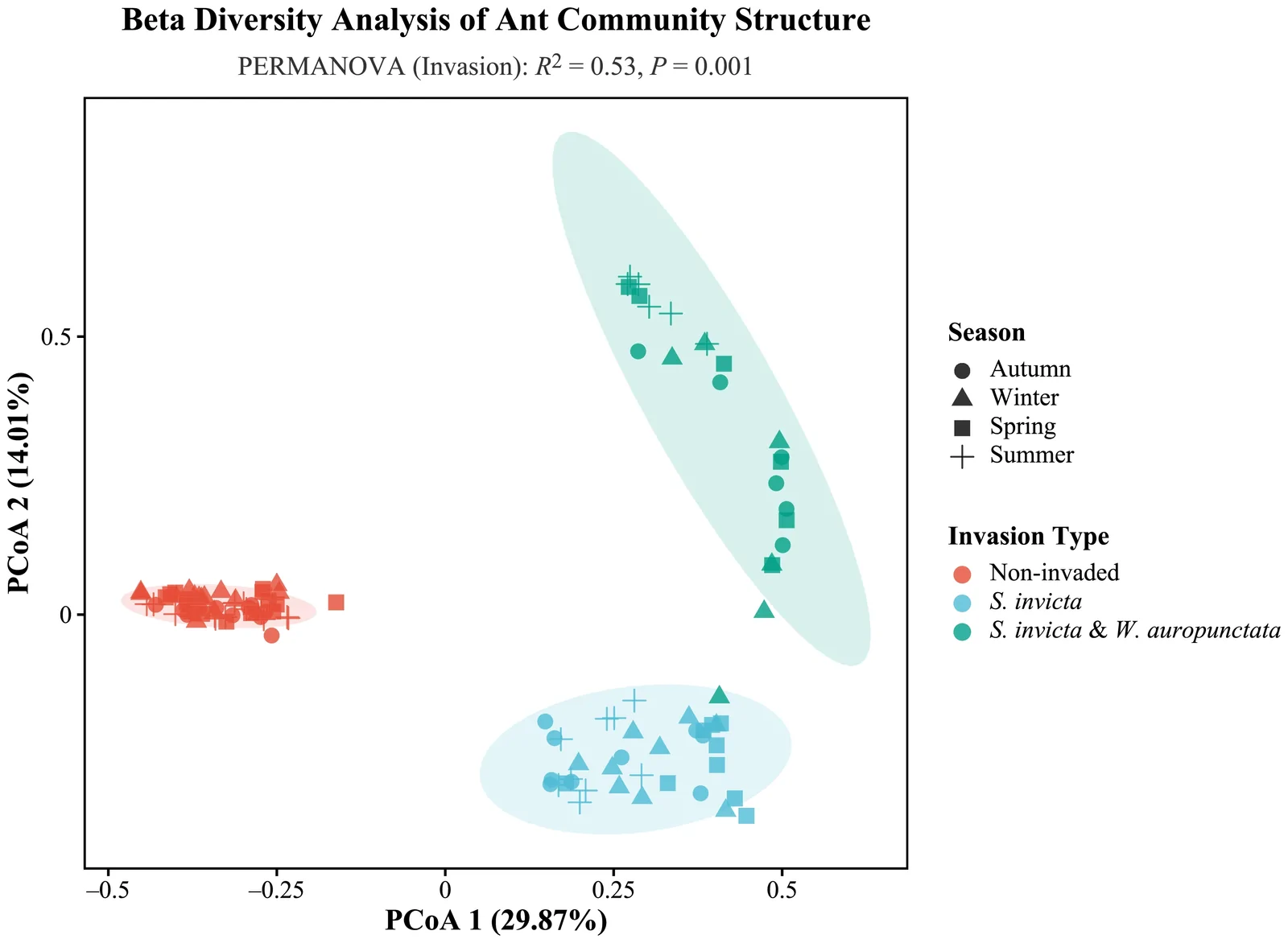
PCoA analysis of native ant community structure in different invaded plots based on temporal dynamic data from Enping City.

To ascertain whether the decline patterns of native communities were driven by temporal dynamics, we evaluated the temporal trajectories of species richness and abundance between the initial and final surveys over the 1-yr observation cycle. After 1 yr, native communities in uninvaded and *S. invicta*-only plots demonstrated high temporal stability, exhibiting only statistically negligible fluctuations (slight increases in richness, minor decreases in abundance; figure 4A–D). By stark contrast, within co-invaded plots, native ant richness and abundance exhibited a significant and persistent downward trajectory over time (figure 4E,F). This decline potentially underscores how the progressive expansion of *W. auropunctata* within these co-invaded landscapes precipitates the eventual collapse of native communities, effectively overcoming the transient buffering effect initially provided by inter-invader antagonism.

**Figure 4.**
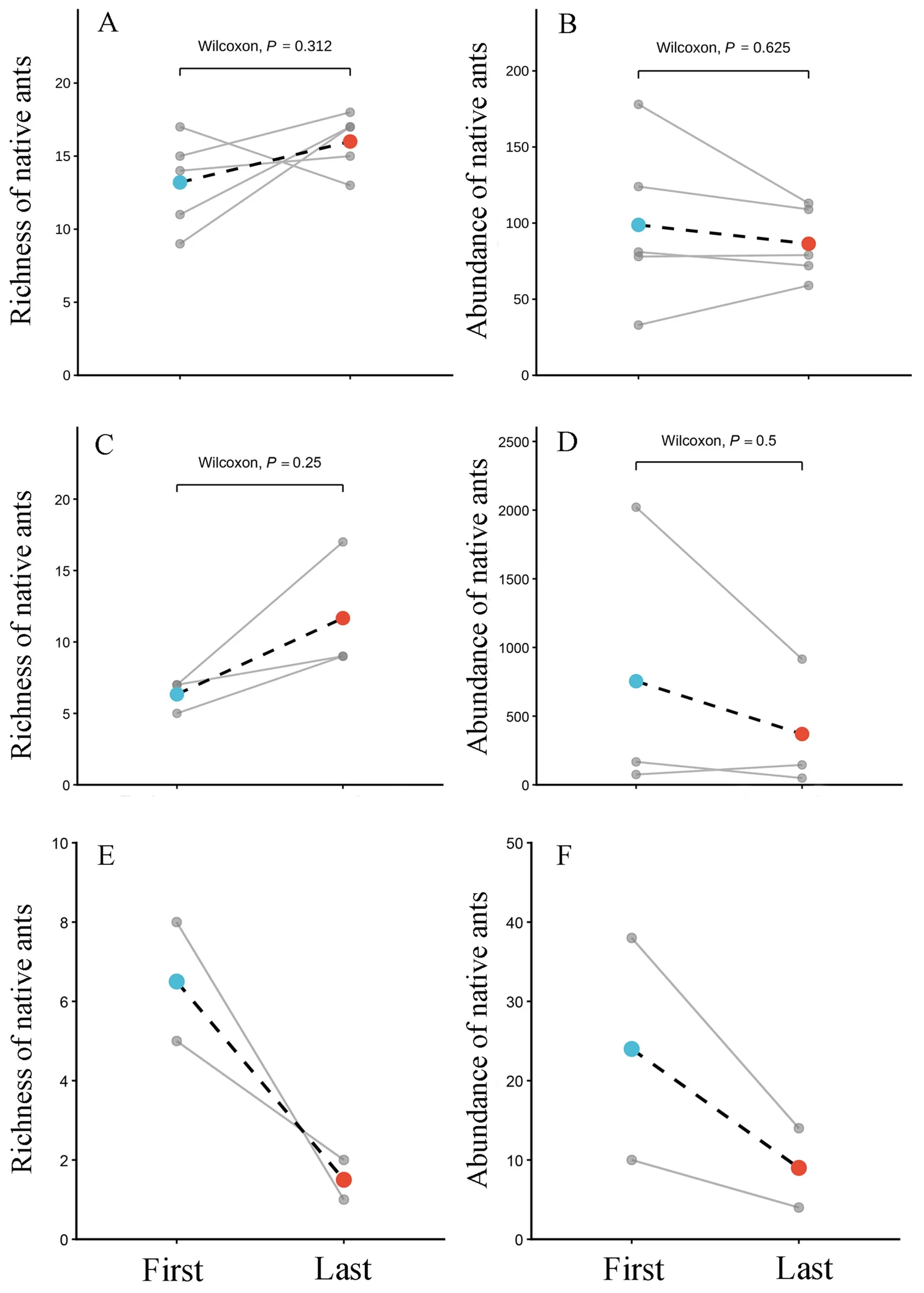
Temporal shifts in species richness (A,C,E) and abundance (B,D,F) of native ant communities from the first to the last survey. (A,B) Non-invaded; (C,D) *S. invicta* invaded; (E,F) *S. invicta* and *W. auropunctata* co-invaded. Small grey dots and connecting lines represent raw data and temporal trajectories of individual replicate plots, respectively. Large coloured circles represent mean values and black dashed lines show the overall trend. Statistical significance between the first and last surveys was determined via Wilcoxon rank-sum tests (*α* = 0.05), with exact *P*-values displayed above the comparisons. Note, owing to the limited number of co-invaded plots (*n* = 2), no *P*-values are shown for (E,F).

### (c) Competitive exclusion dynamics and environment-independent community suppression

Within the co-invaded areas, the relative abundances of *S. invicta* and *W. auropunctata* remained in a state of dynamic oscillation prior to May 2025. However, thereafter (June–August), *S. invicta* was decisively relegated to a competitively inferior position, showing a progressive population decline and ultimately being nearly competitively excluded by *W. auropunctata* (figure 5; supplementary material, figure S3). At this stage, the divergence in abundance between the two invasive ants reached highly significant levels (supplementary material, figure S4). Concurrently, although native ant species richness in this region fluctuated with seasonal temperature variations, the richness in the summer of 2025 (June–August) was significantly lower than in the preceding autumn (September–November 2024), despite similar temperature gradients. This temporal asymmetry indicates that the native community is undergoing persistent ecological degradation, independent of pure temperature effects.

**Figure 5.**
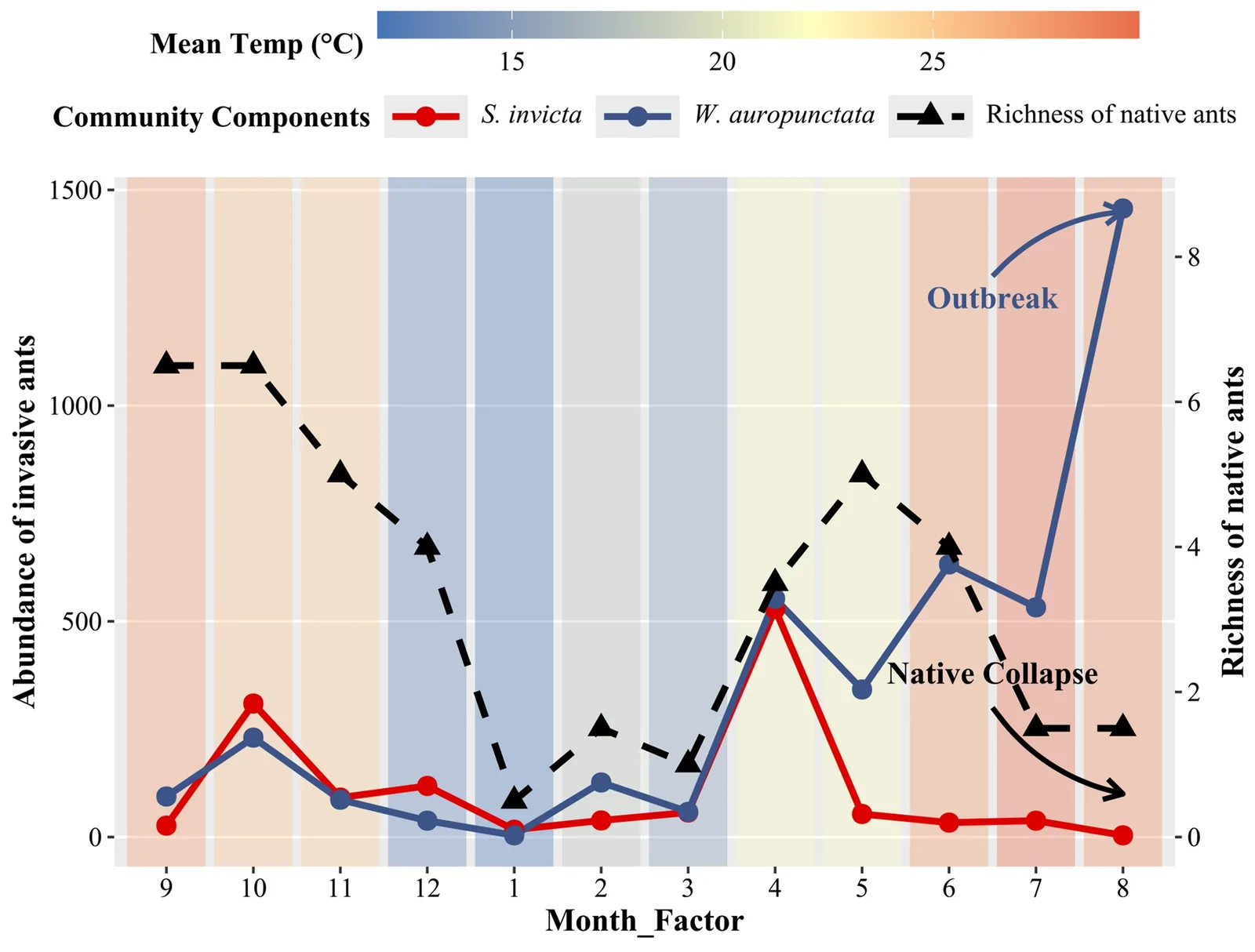
Temporal trajectories of invasive ant populations (*S. invicta* and *W. auropunctata*) and native ant species richness across varying monthly mean temperatures. Sampling months are shown on the *x*-axis. The left and right *y*-axes represent invasive ant population size and native ant species richness, respectively. The background features a heatmap based on mean monthly temperatures.

The GLMM analysis revealed that among the evaluated abiotic covariates, mean temperature was the only factor that significantly and positively influenced both native ant richness and abundance (figure 6). By contrast, other environmental variables—including precipitation, relative humidity, wind speed and atmospheric pressure—showed no statistically significant effects on the native community, as their standardized estimates remained near zero with 95% confidence intervals crossing the significance threshold (figure 6). These results indicate that within our study period, the disassembly of native ant communities was primarily driven by invasive species density and temperature fluctuations, rather than other short-term meteorological variations. Furthermore, interaction analyses revealed that the suppressive effects of both *S. invicta* and *W. auropunctata* on native communities were not significantly modulated by temperature fluctuations (supplementary material, figure S5). This further implies that the invader-dominated community suppression mechanism operates independently of changes in the thermal environment.

**Figure 6.**
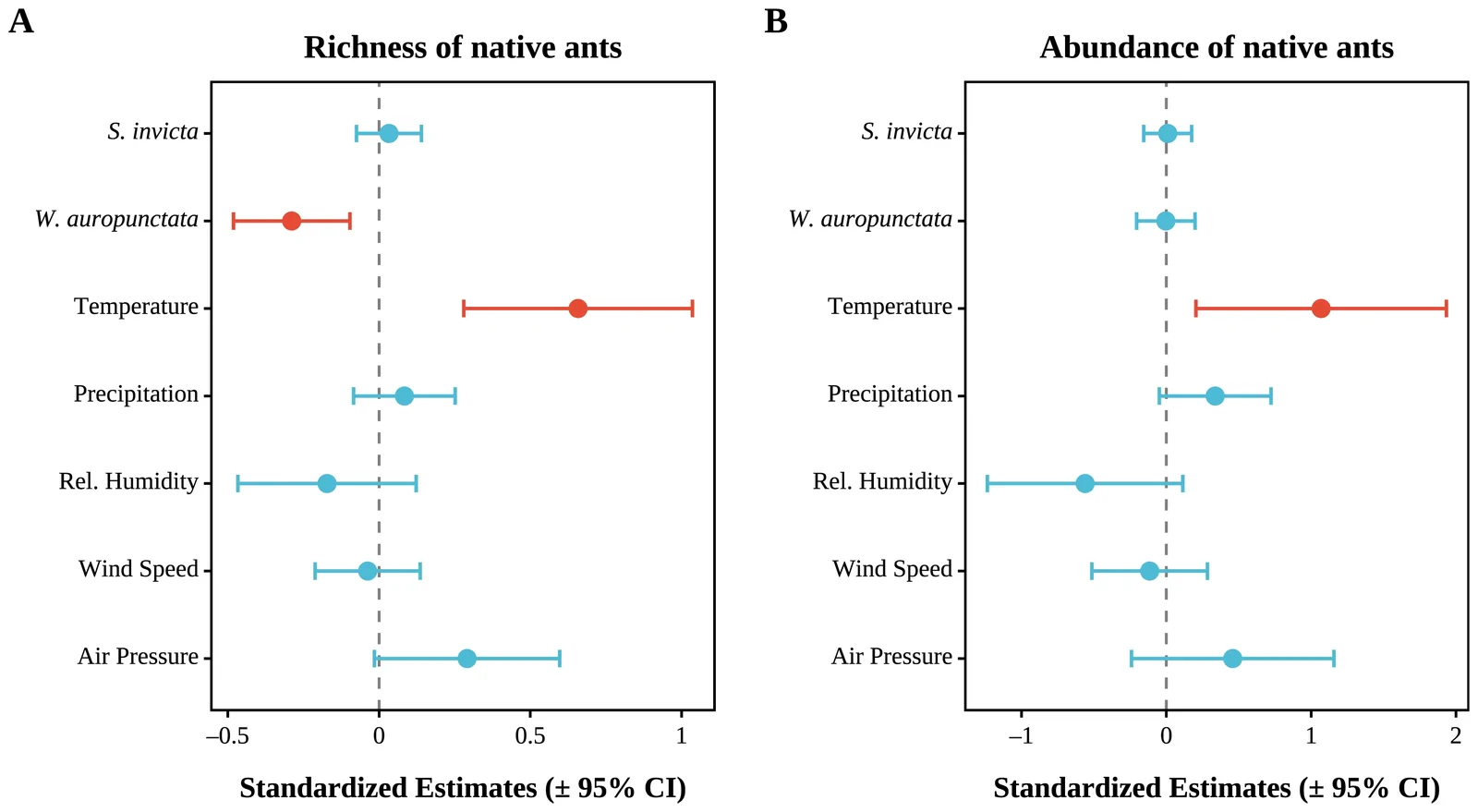
Effects of invasive ant abundance and environmental factors on native ant species richness (A) and abundance (B) based on generalized linear mixed models (GLMMs).

### (d) Thermal responses and behavioural mechanisms driving competitive advantage

Analyses based on year-round temperature data unveiled starkly divergent thermal response patterns between the two invasive ants. Although the overall population abundances of both species exhibited a positive trend with rising temperatures, the population dynamics of *S. invicta* lacked a statistically significant response to temperature fluctuations; this characteristic remained consistent across all spatial distribution plots (single invasion, co-invasion and total invasion plots). Conversely, the population abundance of *W. auropunctata* displayed highly significant temperature dependency (figure 7, p<0.001; $R_{m}^{2}=0.67,R_{c}^{2}=0.71$).

**Figure 7.**
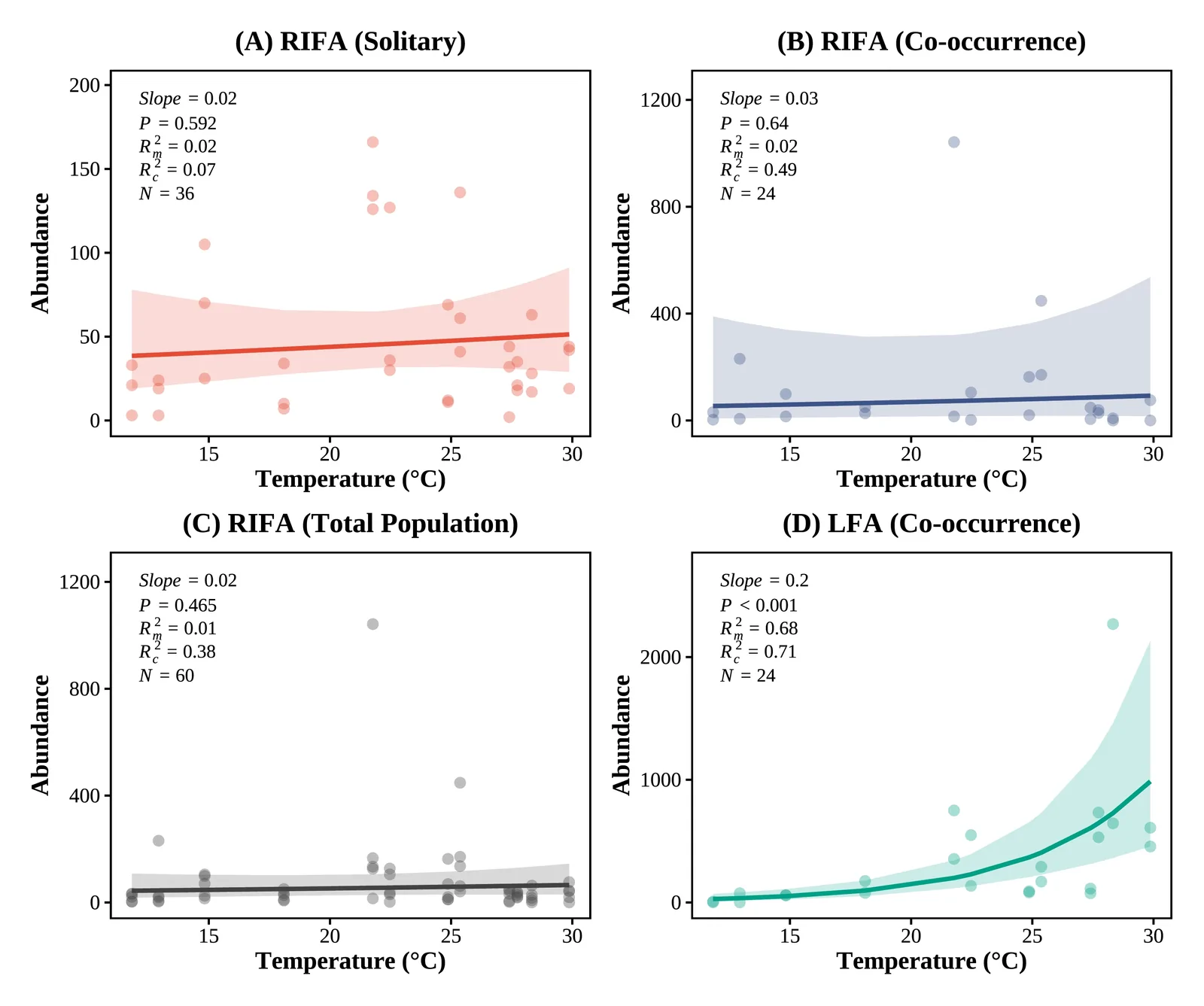
Population responses of *S. invicta* and *W. auropunctata* to temperature variation. (A) Response of *S. invicta* in *S. invicta*-only invaded plots. (B) Response of *S. invicta* in co-invaded plots. (C) Response of *S. invicta* across all invaded plots. (D) Response of *W. auropunctata* in co-invaded plots.

Preliminary experiments demonstrated that out of 151 cumulative encounters between *S. invicta* and *W. auropunctata* across 36 replicates, intense aggressive behaviours erupted only nine times—all initiated by *W. auropunctata*. In all other instances, aggression was initiated by *S. invicta*, but *W. auropunctata* responded with death-feigning (thanatosis) behaviour. Further indoor experiments revealed sharply contrasting survival outcomes between the secondary invader (*W. auropunctata*) and the native control species (*S. jacoti*) under simulated asymmetric interference competition. In scenarios mimicking early-stage invasion, the native *S. jacoti* suffered rapid and devastating casualties, with 60-min mortality rates approaching 1.0 under all density gradients of *S. invicta* attacks (figure 8A). Strikingly, under identical extreme competitive pressure, a single *W. auropunctata* worker demonstrated remarkable survival capacity. Throughout the entire 60-min contact period, the mortality rate of *W. auropunctata* remained exceptionally low (<15%) and this robust survival advantage was virtually unaffected by the attack density of *S. invicta* (figure 8A). This massive survival disparity provides compelling evidence that the ‘death-feigning’ behaviour of *W. auropunctata* possesses outstanding ecological efficacy in evading lethal physical attacks.

**Figure 8.**
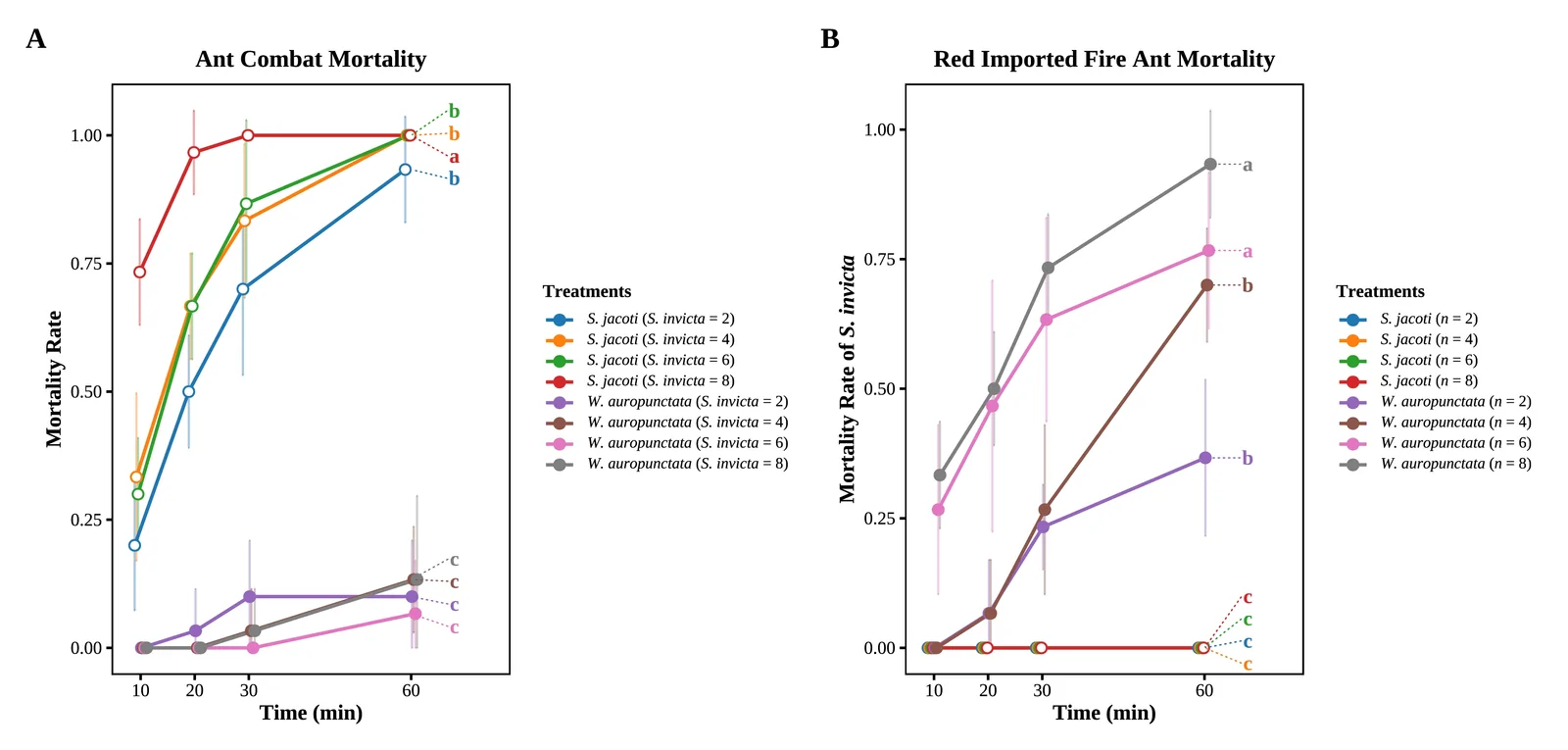
Survival dynamics under asymmetric interference competition across simulated stages of invasion succession. (A) Mortality rates of a single *W. auropunctata* individual and a single *S. jacoti* individual when *S. invicta* is numerically dominant (simulated early succession). (B) Mortality rates of a single *S. invicta* individual when *W. auropunctata* and *S. jacoti* are numerically dominant (simulated late succession). Data are presented as mean ± SD. Different lowercase letters indicate significant differences in mortality over the 60-min duration (Kruskal–Wallis test followed by *post-hoc* multiple comparisons, *p* < 0.05).

Conversely, in scenarios simulating the late stage of invasion (i.e. when *W. auropunctata* establishes numerical dominance), the interspecific competitive landscape underwent a fundamental reversal (figure 8B). When the native *S. jacoti* was pitched as a group against a single *S. invicta* worker, it failed to inflict any lethal damage throughout the observation period (mortality rate = 0). However, numerically dominant *W. auropunctata* exhibited fierce lethality, inflicting mortality upon a single *S. invicta* that was highly density- and time-dependent. At the highest density (eight workers) and longest contact time (60 min), *W. auropunctata* caused over 90% mortality in *S. invicta* (figure 8B). Together, these reverse-validation experiments demonstrate that *W. auropunctata* not only possesses a highly effective defensive strategy to survive the initial period of niche overlap, but it also wields formidable offensive capabilities capable of outcompeting the primary invader once numerical dominance is secured.

## 4. Discussion

Although indoor experiments have confirmed the existence of intense interference competition among invasive ants [40,41], macro-scale field evidence elucidating how multiple invaders reshape native communities remains scarce. The classic ‘invasional meltdown’ hypothesis posits that the coexistence of multiple invasive species triggers synergistic destructive effects [10]. However, leveraging our instantaneous sampling surveys and the additive null model, this study provides compelling evidence that the interaction trajectory between *S. invicta* and *W. auropunctata* during their initial coexistence is heavily skewed toward antagonism rather than synergism (figure 1). This robust interspecific antagonism partially ameliorates their combined exploitation of native resources, unexpectedly providing a buffer zone for native ant communities. This finding suggests that the ‘invasional meltdown’ hypothesis may lack universal applicability among ants, aligning with recent theoretical predictions that antagonistic interactions between co-invaders can actually mitigate ecosystem degradation [14].

Nevertheless, confining ecological conclusions to instantaneous snapshot data is often deceptive. Our longitudinal time-series data reveal that the community buffering generated by the aforementioned antagonistic interactions is merely a process of ‘transient dynamics’ [42]. Temporal trajectories clearly illustrate that as *W. auropunctata* progressively displaces *S. invicta*, the native community is irreversibly driven toward local extirpation (figure 4). This highlights the severe limitations of cross-sectional surveys in capturing the ultimate ‘ecological endgame’ of multiple-invader conflicts. In co-invaded areas, native species richness and abundance experienced precipitous declines, and the GLMM explicitly identified *W. auropunctata* as the core engine driving this community disassembly (figure 6). Notably, *S. invicta* did not exhibit a significant main effect on native ant richness or abundance in the current GLMM. This lack of significance suggests that, within our specific study area and during the observation period, *S. invicta* may not have reached a sufficient density threshold to exert the severe suppressive impacts typically associated with its invasion elsewhere. Such a pattern highlights the context dependency of invasion impacts, where local biotic resistance or specific environmental conditions in these newly colonized sites may constrain the ecological influence of an otherwise dominant invader. This further corroborates that the suppression of native communities by invasive ants operates under a strong density-dependent threshold effect [25,43].

Previous studies have captured phenomena where *S. invicta* is replaced by other dominant ant species (e.g. *Nylanderia fulva*) in localized habitats [22]; yet, the abiotic limiting factors driving such competitive displacement and their consequences have remained a black box. Our research reveals that, although the mechanisms by which these two major invaders suppress native communities are independent of real-time temperature fluctuations, their own absolute population expansions exhibit starkly divergent thermal responses (figure 7). *W. auropunctata* demonstrates a highly robust positive temperature dependency, forming a distinct niche differentiation from the thermal insensitivity of *S. invicta*. While global species distribution models have projected a general reduction in the suitable range for *W. auropunctata* by 2080 under future climatic scenarios, these projections exhibit significant regional heterogeneity, with suitability potentially increasing in certain locales [44]. Our findings align with recent localized studies suggesting that the suitable habitat range in China may actually be expanding [45], underscoring that regional warming can act as an ecological catalyst for this thermophilic invader. Placed within the macro-context of contemporary climate change, this finding portends an alarming ecological trajectory: in multi-invasion networks, *S. invicta* may merely play the role of a ‘pioneer species’ that breaches the defences of native communities, whereas the thermophilic *W. auropunctata* is primed to step in as the ultimate ‘apex invader.’ This implies that as global temperatures continue to rise, climate warming will act as an asymmetric ecological catalyst, significantly amplifying the community-suppressive power of the thermophilic *W. auropunctata*.

Mechanistically, how can the diminutive *W. auropunctata* subvert and replace the highly aggressive, apex invader *S. invicta*? This study unveils a striking paradox of interference competition. Previous works on invasive ant interaction networks have emphasized that behavioural dominance and aggression strategies heavily shape community organization [46]. However, when focusing on physical exclusion, the ultimate winner of these interactions is often determined by physiological resilience and confrontation-induced mortality [38]. We found that the success of *W. auropunctata* is rooted in its highly flexible ‘state-dependent behavioural plasticity.’ During the early succession phase, when it is at a density disadvantage, *W. auropunctata* employs a sophisticated ‘death-feigning’ (thanatosis) behaviour to largely circumvent lethal physical attacks from high-density *S. invicta* (figure 8A). This micro-scale defensive strategy macroscopically buys it a crucial temporal window to traverse the period of niche overlap, ensuring initial colonization and robust population accumulation. Once it breaches a critical abundance threshold and establishes numerical dominance, *W. auropunctata* rapidly shifts into a highly destructive group-attack mode, executing indiscriminate competitive exclusion against both *S. invicta* and native species (figure 8B). However, whether *W. auropunctata* can spatially homogenize across all microhabitats to achieve the complete eradication of native ants, and to what extent its massive population size relies on its unique clonal reproductive advantages [47], remain critical scientific questions that must be urgently addressed to decipher multi-invasion network dynamics.

One consideration when interpreting the joint fluctuations of worker abundance and native species richness may relate to the species-specific social organization and colony traits of these invasive ants. Owing to the non-destructive nature of our long-term monitoring, direct mapping of subterranean and litter-dwelling colonies was unfeasible, which presents an inherent limitation in separating individual-worker activity from true colony-level demographics. Nonetheless, the contrasting worker abundance trajectories of *S. invicta* and *W. auropunctata* could be linked to their divergent colony-level traits. Both of the species frequently exhibit highly polygynous and unicolonial (or polydomous) social structures, which can facilitate the formation of interconnected, non-aggressive nest networks [25]. For *W. auropunctata*, the observed positive response of worker abundance to seasonal warming might not simply reflect increased individual activity, but could also indicate the localized expansion or cooperative budding of decentralized colony networks. Such expansion could enable a sustained influx of workers into foraging arenas, potentially contributing to the observed decline in native species richness. Conversely, many native ants in these highly disturbed habitats often possess monodomous nesting structures with smaller colony sizes, which might increase their vulnerability to coordinated worker foraging.

This dynamic of competitive exclusion in the introduced range stands in contrast to the patterns observed within their native South American range. In fragmented urban habitats in Argentina, *S. invicta* and *W. auropunctata* co-exist with other globally invasive ants, such as *Linepithema humile* and *N. fulva* [48]. In these native patches, coexistence is mediated by a metacommunity framework characterized by fine-scale spatial structuring and trait-based trade-offs rather than a rigid dominance hierarchy; for instance, while *L. humile* excels at recruitment and *W. auropunctata* displays highly localized dominance restricted near nesting areas, *S. invicta* remains relatively rare and competitively subordinate [48]. By contrast, our study suggests that in newly introduced environments, the absence of these complex, multi-species regulatory networks and spatial buffering allows *W. auropunctata* to leverage its behavioural plasticity and thermal advantages, transitioning from localized persistence to landscape-scale competitive displacement of *S. invicta*.

Based on these mechanisms, this study calls for a fundamental paradigm shift in global biosecurity and conservation strategies. In vulnerable ecosystems undergoing co-invasion, traditional eradication protocols targeting a single high-profile species (e.g. targeted toxic baiting exclusively for *S. invicta*) may not only prove futile but could ignite an ecological disaster. Because *S. invicta* exerts a degree of antagonistic suppression on *W. auropunctata* during the early stages, the rash removal of *S. invicta* could inadvertently trigger the ‘ecological release’ of a far more destructive secondary competitor [49], thereby accelerating the collapse of native biodiversity. To navigate the increasingly complex ecosystems of the Anthropocene, we must abandon single-species prioritization strategies and pivot toward systemic invasion management grounded in multi-species network topologies and mechanistic drivers.

## 5. Conclusion

Our study adds to the growing body of evidence regarding the competitive displacement of apex invaders, demonstrating that when two of the world's most destructive supercolonial ants collide, the outcome is neither a synergistic invasional meltdown nor a stable coexistence. Instead, we reveal a highly asymmetric ‘temporal displacement’ driven by state-dependent behavioural mechanisms, which fundamentally reshapes the trajectory of native community disassembly. By shifting the analytical lens from traditional static ‘ecological snapshots’ to continuous spatiotemporal trajectories, this research provides novel empirical evidence regarding the specific interactions between *S. invicta* and *W. auropunctata*.

## Supplementary Material



## Data Availability

All raw data and code have been uploaded as supplementary materials [50].

## References

[1] Seebens H *et al.* 2017 No saturation in the accumulation of alien species worldwide. Nat. Commun. 8, 14435. (10.1038/ncomms14435) PMC5316856 28198420

[2] Wallingford PD *et al.* 2020 Adjusting the lens of invasion biology to focus on the impacts of climate-driven range shifts. Nat. Clim. Change 10, 398–405. (10.1038/s41558-020-0768-2)

[3] Liu Y , Oduor AM, Zhang Z, Manea A, Tooth IM, Leishman MR, Xu X, Van Kleunen M. 2017 Do invasive alien plants benefit more from global environmental change than native plants? Global Change Biol. 23, 3363–3370. (10.1111/gcb.13579) 27888560

[4] Sorte CJB *et al.* 2013 Poised to prosper? A cross-system comparison of climate change effects on native and non-native species performance. Ecol. Lett. 16, 261–270. (10.1111/ele.12017) 23062213

[5] Crain CM , Kroeker K, Halpern BS. 2008 Interactive and cumulative effects of multiple human stressors in marine systems. Ecol. Lett. 11, 1304–1315. (10.1111/j.1461-0248.2008.01253.x) 19046359

[6] Côté IM , Darling ES, Brown CJ. 2016 Interactions among ecosystem stressors and their importance in conservation. Proc. R. Soc. B 283, 20152592. (10.1098/rspb.2015.2592) PMC4760168 26865306

[7] Kuebbing SE , Nuñez MA, Simberloff D. 2013 Current mismatch between research and conservation efforts: the need to study co-occurring invasive plant species. Biol. Conserv. 160, 121–129. (10.1016/j.biocon.2013.01.009)

[8] Preston DL , Henderson JS, Johnson PT. 2012 Community ecology of invasions: direct and indirect effects of multiple invasive species on aquatic communities. Ecology 93, 1254–1261. (10.1890/11-1821.1) 22834365

[9] Braga RR , Gómez-Aparicio L, Heger T, Vitule JRS, Jeschke JM. 2018 Structuring evidence for invasional meltdown: broad support but with biases and gaps. Biol. Invasions 20, 923–936. (10.1007/s10530-017-1582-2)

[10] Simberloff D , Von Holle B. 1999 Positive interactions of nonindigenous species: invasional meltdown? Biol. Invasions 1, 21–32. (10.1023/A:1010086329619)

[11] O'Dowd DJ , Green PT, Lake PS. 2003 Invasional ‘meltdown’ on an oceanic island. Ecol. Lett. 6, 812–817. (10.1046/j.1461-0248.2003.00512.x)

[12] D'Antonio CM , Vitousek PM. 1992 Biological invasions by exotic grasses, the grass/fire cycle, and global change. Annu. Rev. Ecol. Syst. 23, 63–87. (10.1146/annurev.es.23.110192.000431)

[13] Li C-W *et al.* 2026 Divergence among species with “good competitor” and “good cultivator” strategies promotes asymmetric facilitation among co-invaders. Nat. Commun. 17, 4688. (10.1038/s41467-026-73076-2) PMC13212657 42140961

[14] Kuebbing SE , Nuñez MA. 2015 Negative, neutral, and positive interactions among nonnative plants: patterns, processes, and management implications. Global Change Biol. 21, 926–934. (10.1111/gcb.12711) 25142018

[15] Tekiela DR , Barney JN. 2017 Co-invasion of similar invaders results in analogous ecological impact niches and no synergies. Biol. Invasions 19, 147–159. (10.1007/s10530-016-1269-0)

[16] Kideys AE . 2002 Fall and rise of the Black Sea ecosystem. Science 297, 1482–1484. (10.1126/science.1073002) 12202806

[17] MacArthur R , Levins R. 1967 The limiting similarity, convergence, and divergence of coexisting species. Am. Nat. 101, 377–385. (10.1086/282505)

[18] Reitz SR , Trumble JT. 2002 Competitive displacement among insects and arachnids. Annu. Rev. Entomol. 47, 435–465. (10.1146/annurev.ento.47.091201.145227) 11729081

[19] Bargielowski IE , Lounibos LP, Carrasquilla MC. 2013 Evolution of resistance to satyrization through reproductive character displacement in populations of invasive dengue vectors. Proc. Natl. Acad. Sci. USA 110, 2888–2892. (10.1073/pnas.1219599110) PMC3581888 23359710

[20] Pearson DE , Ortega YK, Eren Ö, Hierro JL. 2016 Quantifying ‘apparent’ impact and distinguishing impact from invasiveness in multispecies plant invasions. Ecol. Appl. 26, 162–173. (10.1890/14-2345) 27039517

[21] Strayer DL , Eviner VT, Jeschke JM, Pace ML. 2006 Understanding the long-term effects of species invasions. Trends Ecol. Evol. 21, 645–651. (10.1016/j.tree.2006.07.007) 16859805

[22] LeBrun EG , Abbott J, Gilbert LE. 2013 Imported crazy ant displaces imported fire ant, reduces and homogenizes grassland ant and arthropod assemblages. Biol. Invasions 15, 2429–2442. (10.1007/s10530-013-0463-6)

[23] Strayer DL . 2017 Boom-bust dynamics in biological invasions: towards an improved application of the concept. Biol. Rev. 92, 2051–2068. (10.1111/ele.12822) 28834087

[24] LeBrun EG , Jones NT, Gilbert LE. 2014 Chemical warfare among invaders: a detoxification interaction facilitates an ant invasion. Science 343, 1014–1017. (10.1126/science.1245833) 24526314

[25] Holway DA , Lach L, Suarez AV, Tsutsui ND, Case TJ. 2002 The causes and consequences of ant invasions. Annu. Rev. Ecol. Syst. 33, 181–233. (10.1146/annurev.ecolsys.33.010802.150444)

[26] Lach L , Hooper‐Bùi LM. 2010 Consequences of ant invasion. In Ant ecology. (eds Lach L, Parr C, Abbott K), pp. 261–286. Oxford, UK: Oxford University Press. (10.1093/acprof:oso/9780199544639.003.0015)

[27] Xu Y , Vargo EL, Tsuji K, Wylie R. 2022 Exotic ants of the Asia-Pacific: invasion, national response, and ongoing needs. Annu. Rev. Entomol. 67, 27–42. (10.1146/annurev-ento-060721-085603) 34582264

[28] Lowe S , Browne M, Boudjelas S, De Poorter M. 2000 100 of the world's worst invasive alien species: a selection from the global invasive species database, Vol. 12. pp. 12. Auckland, New Zealand: Invasive Species Specialist Group.

[29] Clark DB , Guayasamin C, Pazmino O, Donoso C, de Villacis YP. 1982 The tramp ant *Wasmannia auropunctata*: autecology and effects on ant diversity and distribution on Santa Cruz Island, Galapagos. Biotropica 14, 196–207. (10.2307/2388026)

[30] Porter SD , Savignano DA. 1990 Invasion of polygyne fire ants decimates native ants and disrupts arthropod community. Ecology 71, 2095–2106. (10.2307/1938623)

[31] Le Breton J , Delabie JHC, Chazeau J, Dejean A, Jourdan H. 2004 Experimental evidence of large-scale unicoloniality in the tramp ant *Wasmannia auropunctata* (Roger). J. Insect Behav. 17, 263–271. (10.1023/B:JOIR.0000028575.28700.71)

[32] Tschinkel WR . 2013 The fire ants. Cambridge, UK: Belknap Press. (10.2307/j.ctv1dp0txz)

[33] Gotelli NJ , Arnett AE. 2000 Biogeographic effects of red fire ant invasion. Ecol. Lett. 3, 257–261. (10.1046/j.1461-0248.2000.00138.x)

[34] Ivanov K , Keiper J. 2009 Effectiveness and biases of Winkler litter extraction and pitfall trapping for collecting ground-dwelling ants in northern temperate forests. Environ. Entomol. 38, 1724–1736. (10.1603/022.038.0626) 20021769

[35] Lopes CT , Vasconcelos HL. 2008 Evaluation of three methods for sampling ground-dwelling ants in the Brazilian Cerrado. Neotrop. Entomol. 37, 399–405. (10.1590/S1519-566X2008000400007) 18813741

[36] Hu J , Bogar TA, Hamer MT, Guénard B. 2025 Impacts of single and multiple co-existing invasive species on subtropical native ant communities. Ecol. Evol. 15, e72095. (10.1002/ece3.72095) PMC12401564 40900727

[37] Zhao Y , Chen D, Xing Z, He H, Cai Z, Zhao Y, Guénard B, Xu Y. 2026 Co-invasion of multiple invasive ants exacerbates the impacts on native ant communities. Insect Sci. (10.1111/1744-7917.70274) 41873177

[38] Nie L , Ni M, Ning D, Ran H, Hassan B, Xu Y. 2019 Comparing mechanisms of competition among introduced and resident ants in China: from behaviour to trophic position (Hymenoptera: Formicidae). Myrmecol. News 29, 125–133. (10.25849/myrmecol.news_029:125)

[39] Chen D , Liao Z, Song J, Mao M, Ke Z, Xu Y. 2026 Changes in the aggressiveness of invasive red imported fire ants towards native ants: perspectives based on different latitudes and invasion status. Myrmecol. News 36, 137–143 (10.25849/myrmecol.news_036:137)

[40] Bentley MT , Hahn DA, Oi FM. 2016 The thermal breadth of *Nylanderia fulva* (Hymenoptera: Formicidae) is narrower than that of *Solenopsis invicta* at three thermal ramping rates: 1.0, 0.12, and 0.06 C min^−1^. Environ. Entomol. 45, 1058–1062. (10.1093/ee/nvw050) 27252409

[41] Nie L , Bamisile BS, Li Y, Ran H, Wang S, Wang L, Xu Y. 2023 Interspecific competition predicts the potential impact of little fire ant *Wasmannia auropunctata* (Roger) (Hymenoptera: Formicidae) invasion on resident ants in southern China. Insect Sci. 30, 1518–1530. (10.1111/1744-7917.13170) 36583375

[42] Hastings A *et al.* 2018 Transient phenomena in ecology. Science 361, eaat6412. (10.1126/science.aat6412) 30190378

[43] Calcaterra LA , Livore JP, Delgado A, Briano JA. 2008 Ecological dominance of the red imported fire ant, *Solenopsis invicta*, in its native range. Oecologia 156, 411–421. (10.1007/s00442-008-0997-y) 18305962

[44] Bertelsmeier C , Luque GM, Hoffmann BD, Courchamp F. 2015 Worldwide ant invasions under climate change. Biodivers. Conserv. 24, 117–128. (10.1007/s10531-014-0794-3)

[45] Wang H , Sui X, Wang X, Xiao J, Gao X, Wang H. 2024 Invasion alert for *Wasmannia auropunctata*: a potential distribution in southern China and associated invasion risk to urban, rural, agricultural, and forest lands. J. Pest Sci. 97, 597–612. (10.1007/s10340-023-01694-6)

[46] Trigos-Peral G , Abril S, Angulo E,. 2021 Behavioral responses to numerical differences when two invasive ants meet: the case of *Lasius neglectus* and *Linepithema humile*. *Biol. Invasions* 23, 955. (10.1007/s10530-020-02439-7)

[47] Foucaud J , Orivel J, Fournier D, Delabie JHC, Loiseau A, Le Breton J, Cerdans P, Estoup A. 2009 Reproductive system, social organization, human disturbance and ecological dominance in native populations of the little fire ant, *Wasmannia auropunctata*. Mol. Ecol. 18, 5059–5073. (10.1111/j.1365-294X.2009.04440.x) 19943889

[48] Muñoz IJ , Alvarez Costa A, Schilman PE, Calcaterra LA. 2026 Spatial coexistence of invasive ants in fragmented urban habitats of their native range. Front. Insect Sci. 6, 1776153. (10.3389/finsc.2026.1776153) PMC12971952 41815923

[49] Zavaleta ES , Hobbs RJ, Mooney HA. 2001 Viewing invasive species removal in a whole-ecosystem context. Trends Ecol. Evol. 16, 454–459. (10.1016/S0169-5347(01)02194-2)

[50] Chen D , Liao Z, Song J, Ke Z, Mao M, Li S, Lu Y, Xu Y. 2026 Supplementary material from: Thermal asymmetry and density-dependent behavioural shifts drive competitive displacement between two globally invasive ants. Figshare. (10.6084/m9.figshare.c.8675504) PMC13751126 43008824

