# Supplementary material for: Thermal asymmetry and density-dependent behavioural shifts drive competitive displacement between two globally invasive ants
Source: Proc Biol Sci. 2026 Nov 11;293(2082):20261951. doi: 10.1098/rspb.2026.1951 (PMC13751126; doi:10.1098/rspb.2026.1951)
Supplement: Supplementary file 1 [file rspb.2026.1951.zip › rspb.2026.1951/rspb20261951_si_001.docx]

Thermal asymmetry and density-dependent behavioral shifts drive competitive displacement between two globally invasive ants

Defu Chen^1^, Zhuoquan Liao^1^, Jiale Song^1^, Zengyuan Ke^1^, Mengfei Mao^1^, Shaopeng Li^2^, Yongyue Lu^1^, Yijuan Xu^1*^

1 Red Imported Fire Ant Research Center, South China Agricultural University, Guangzhou 510642, China

2 School of Ecological and Environmental Sciences, East China Normal University, Shanghai, China.

*Correspondence: Yijuan Xu ([xuyijuan@yahoo.com](mailto:xuyijuan@yahoo.com)).

Journal name: Proceedings of the Royal Society B

Article DOI: 10.1098/rspb.2026.1951

**Supplementary Materials and Methods**

**1 Study Sites, Ant Collection, and Environmental Factors**
To investigate the potential interspecific interactions and invasion dynamics among invasive ant species, this study integrated two strategies: a spatial snapshot survey and longitudinal dynamic monitoring. First, in August 2024, a spatial snapshot survey was conducted in Shantou City, Guangdong Province. A total of 48 plots were randomly selected, comprising 3 uninvaded plots, 15 plots invaded solely by the red imported fire ant (*S. invicta*), 18 plots invaded solely by the little fire ant (*W. auropunctata*), and 12 plots co-invaded by both species. To ensure spatial independence while capturing landscape-scale variation, the inter-plot distances for the 48 spatial snapshot plots in Shantou ranged from a minimum of 100 m to a maximum of 23 km. All selected plots shared a similar habitat type—heavily anthropogenically disturbed urban greenbelts. Furthermore, a one-year longitudinal dynamic monitoring program was carried out from September 2024 to August 2025 in Enping City, Guangdong Province. For the 10 longitudinal monitoring plots in Enping, inter-plot distances ranged between 100 m and 30 km. This monitoring included 10 plots situated in mixed agricultural and wasteland habitats (5 uninvaded, 3 solely invaded by *S. invicta*, and 2 co-invaded plots). Notably, Enping City was selected as the time-series monitoring site based on our baseline census conducted in 2023 (manuscript under review). The census revealed that this region represents a newly colonized area for *W. auropunctata* with low initial population densities, providing an ideal natural system to study the early dynamics of invasion between two invasive ants under natural conditions.

Each study plot measured 12 m × 9 m. To comprehensively characterize the local ant community, we employed a standardized sampling strategy combining pitfall trapping (Gotelli & Arnett, 2000) and Winkler extraction (Lopes & Vasconcelos, 2008; Ivanov & Keiper, 2009). Specifically, 12 pitfall traps (plastic cups: 4.8 cm bottom diameter, 6.45 cm top diameter, 7 cm height, ~175 mL capacity) were deployed in each plot following a standardized grid layout designed for subtropical invasive ant assessments (Hu et al., 2025; Zhao et al., 2026). The rim of each cup was set flush with the soil surface, and the cups were filled to one-third of their volume with a 1:1 mixture of absolute ethanol and propylene glycol. Traps were collected after 48 hours. Litter collection and Winkler extraction strictly followed the standardized protocols of Lopes and Vasconcelos (2008). Samples collected via both methods from the same plot were pooled to represent the ant community composition of that specific site.

To control for the potential confounding effects of real-time weather conditions on sampling, daily meteorological data during the sampling periods (including mean temperature, precipitation, relative humidity, and atmospheric pressure) were obtained from the China Meteorological Data Service Centre ([http://data.cma.cn/](https://www.google.com/url?sa=E&q=http%3A%2F%2Fdata.cma.cn%2F)).

**2 Design of Indoor Asymmetric Interference Competition Experiments**
This study posits that during the invasion process, the ability of *W. auropunctata*—a secondary invader—to successfully colonize areas dominated by high-density *S. invicta* is primarily attributable to its unique behavioral strategies. Specifically, during inevitable interference competition, *W. auropunctata* significantly prolongs its survival by exhibiting “death-feigning” (thanatosis) behavior. To validate this hypothesis from a behavioral mechanism perspective, we designed a series of indoor aggression and survival experiments. The design of these asymmetric confrontation trials and the attack index referred to Nie et al. (2019).

First, preliminary one-on-one trials were conducted to evaluate behavioral interactions between the two species. For these assays, we collected 4 independent colonies for each of the three target species (*S. invicta* from Guangzhou; *W. auropunctata* from Enping; *S. jacoti* from Xiaogan). For the invasive species, sampled nests were separated by at least 20 m for *S. invicta* and 10 m for *W. auropunctata* to ensure sampling from physically distinct nest mounds across the site. However, given that both *S. invicta* and *W. auropunctata* predominantly exhibit supercoloniality and unicolonial social structures in their introduced ranges (Holway et al., 2002), these physically separate nests likely shared a supercolonial origin. Based on prior research (Chen et al., 2026) and our preliminary validation, this unicolonial nature intrinsically accounts for the lack of inter-nest behavioral divergence, justifying why colony identity was not treated as a random effect. A single *S. invicta* worker and a single *W. auropunctata* worker were introduced into a Petri dish (5.5 cm diameter). Over a 5-minute period (36 replicates), we focused on the decisive outcomes of contact. While intermediate interactions (avoidance, threat displays) were observed, our analysis specifically quantified the frequency of thanatosis to confirm it as a primary defensive node.

Building on these 1v1 observations, we then established asymmetric confrontation trials to test whether this thanatosis strategy translates into a survival advantage under intense ecological pressure. To simulate the early stage of invasion, where *S. invicta* holds an absolute numerical advantage, we introduced different densities (2, 4, 6, or 8 workers) of *S. invicta* against 1 *W. auropunctata*. The survival status of the *W. auropunctata* worker was recorded at 10, 20, 30, and 60 minutes. We conducted 20 independent replicates per density gradient, divided into 4 batches (5 dishes per batch) to calculate batch mortality rates. To control for body size, the native species *S. jacoti*, which is similar in size but lacks thanatosis, was used in parallel trials following the identical protocol.

Finally, to test the late-stage invasion scenario where *W. auropunctata* achieves numerical superiority, reverse-density validation experiments were conducted. We introduced *W. auropunctata* at densities of 2, 4, 6, or 8 workers against 1 *S. invicta* worker, recording the mortality of *S. invicta* over 60 minutes. *S. jacoti* was again used as a control for *W. auropunctata* to evaluate whether the offensive capacity was unique to the invader. All experimental designs and attack criteria referred to Nie et al. (2019).

**3 Data and Statistical Analysis**
All statistical analyses were performed in R software (version 4.5.2). To evaluate the individual and joint impacts of *S. invicta* and *W. auropunctata* on native ant communities, we first utilized the Wilcoxon rank-sum test to analyze differences in both the spatial snapshot and longitudinal time-series data. Multidimensional dissimilarities in community composition across different invasion scenarios were subjected to dimensionality reduction and visualized using Principal Coordinates Analysis (PCoA). To further elucidate changes in the abundance of *S. invicta* and *W. auropunctata* across different stages, the survey periods were categorized by season, and the Wilcoxon rank-sum test was applied to determine significance. Mortality data from the indoor experiments were treated as count data and analyzed using the Wilcoxon rank-sum test.

**Additive Null Model for Verifying Interaction Effects**
To rigorously determine whether the interactions between the two invasive ants resulted in additive, synergistic, or antagonistic effects, an additive null model was constructed. Assuming the ecological impacts of the two invasive species are independent, the expected mean ($\mu_{exp}$) for the co-invasion group was defined as:

$\mu_{exp}=\max(0,\mu_{A}+\mu_{B}-\mu_{C})$,

where $\mu_{A}$, $\mu_{B}$, and $\mu_{C}$ represent the observed means for the *S. invicta*-only invaded, *W. auropunctata*-only invaded, and uninvaded groups, respectively (the lower bound was set to 0 to align with biological reality). The expected standard error ($SE_{exp}$) was calculated based on the error propagation formula:

$SE_{exp}=\sqrt{SE_{A}^{2}+SE_{B}^{2}+SE_{C}^{2}}$.

Subsequently, a one-sample Wilcoxon signed-rank test was employed to compare the observed values of the co-invasion group with the expected means. Interaction effects were classified based on the following criteria: (1) Additive effect: no significant difference ($P\geq0.05$); (2) Antagonistic effect: significant difference ($P<0.05$) with observed values higher than expected, indicating diminished joint destructive impact; (3) Synergistic effect: significant difference ($P<0.05$) with observed values lower than expected, indicating amplified destructive impact.

**Temporal Trajectories and Generalized Linear Mixed Models (GLMMs)**
To visually demonstrate the phenology and dynamic trajectories of invasive species and native ant communities under evolving temperature conditions, we constructed a multidimensional time-series visualization framework. Considering the natural emergence and outbreak cycles of the target invasive ants, the sampling months were rearranged into an “ecological year” spanning from September to August of the following year. Within this framework, the monthly mean abundances of both invasive ants and the species richness of the native ant community were tracked simultaneously. To address inherent scaling differences between invasive ant abundance and native ant richness, native ant richness data were linearly transformed using specific scaling coefficients and precisely mapped onto a secondary Y-axis, enabling the joint presentation of multiple components in a single figure. Simultaneously, continuous changes in mean environmental temperature were integrated as a background gradient layer (heatmap) to intuitively elucidate the coupling relationship between population dynamics and temperature fluctuations. Key ecological inflection points (e.g., “Outbreak” and “Native Collapse”) were explicitly annotated in the model to highlight critical periods of biological interactions.

Generalized Linear Mixed Models (GLMMs) were constructed using the *glmmTMB* package in R to evaluate the effects of invasive ants (*S. invicta* and *W. auropunctata*) and environmental factors on native ant communities. To control for confounding abiotic influences, we included Mean Temperature, Precipitation, Relative Humidity, Wind Speed, and Atmospheric Pressure as fixed-effect covariates. All continuous predictor variables were Z-score standardized prior to modeling to ensure the comparability of regression coefficients and facilitate direct comparison of relative effect sizes. Although ant activity often exhibits non-linear thermal responses, preliminary model comparisons using natural splines (df = 2) and polynomial terms did not significantly improve model fit ($\Delta AIC<2$) compared to linear predictors; thus, linear terms were retained for parsimony and clearer interpretability. Native ant species richness was fitted with a Poisson distribution, while abundance followed a negative binomial distribution (nbinom2) to account for data overdispersion. To address the nested experimental design and temporal autocorrelation, plot and sampling date were included as random intercepts. We first fitted models to assess the independent effects of each variable and subsequently tested for interactions between invasive ant density and temperature. Model convergence was verified via the Hessian matrix, and statistical significance was determined based on whether the 95% confidence intervals of the standardized estimates crossed zero.

To quantitatively analyze the response patterns of invasive ant abundance to environmental temperature fluctuations, statistical modeling via GLMMs (based on the *glmmTMB* package) was applied. All models were fitted using a negative binomial error distribution (*nbinom2* package). In the model structure, temperature was set as a continuous fixed effect; simultaneously, to account for spatial non-independence among sampling sites and statistical biases from repeated measures, plot was included as a random intercept. Independent GLMMs were constructed for the abundance of *S. invicta* in solely invaded areas, co-invaded areas, and total invaded areas, as well as for *W. auropunctata* in co-invaded areas. Goodness-of-fit was assessed using Nakagawa's pseudo$R^{2}$ tailored for mixed models, reporting both marginal $R^{2}$ ($R_{m}^{2}$) and conditional $R^{2}$ ($R_{c}^{2}$). To visualize the model outputs intuitively, marginal predicted means and their 95% confidence intervals (excluding random effects) were extracted using the *ggeffects* package, generating fitted trajectory curves for temperature responses.

**Figure S1** Comparison of native ant richness in different invaded areas across seasons in Enping City (in chronological order).

**Figure S2** Comparison of native ant abundance in different invaded areas across seasons in Enping City (in chronological order).

**Figure S3** Annual dynamic trends in the relative abundance of *S. invicta* and *W. auropunctata*

**Figure S4** Comparison of the abundance of *S. invicta* and *W. auropunctata* across seasons in co-invaded areas.

**Figure S5** Results of the GLMM based on the interaction between invasive ant species and temperature.
